# Case Report: Primary squamous cell carcinoma of the pancreatic segment of the extrahepatic bile duct: a rare tumor easily misdiagnosed as pancreatic malignancy

**DOI:** 10.3389/fonc.2026.1828272

**Published:** 2026-05-20

**Authors:** Ting Xu, Xue Meng, Xuan Gou, Xinyuan Wang, Shuai Luo, Yilin Chen

**Affiliations:** 1 Department of Pathology, Zhejiang Provincial People's Hospital Bijie Hospital (The First People's Hospital of Bijie), Bijie, Guizhou, China; 2 Medical Imaging Department, Zhejiang Provincial People's Hospital Bijie Hospital (The First People's Hospital of Bijie), Bijie, Guizhou, China; 3 Department of Pathology, Affiliated Hospital of Zunyi Medical University, Zunyi, Guizhou, China

**Keywords:** clinicopathologic features, extrahepatic common bile duct, pancreatic segment of the common bile duct, squamous cell carcinoma, treatment

## Abstract

**Background:**

Cholangiocarcinoma is a malignant tumor arising from the bile duct epithelium and may occur anywhere along the biliary tree, from intrahepatic ducts to the distal common bile duct. Most bile duct malignancies are histologically adenocarcinomas, whereas squamous cell carcinoma (SCC) is exceedingly rare. Herein, we report a case of extrahepatic bile duct SCC arising in the pancreatic segment, which is prone to misdiagnosis as pancreatic cancer.

**Case presentation:**

A 70-year-old male patient with no history of malignancy presented with worsening right upper abdominal pain and postprandial jaundice that worsened after meals, and underwent percutaneous transhepatic biliary drainage. The patient was clinically stable. Subsequently, he underwent open radical pancreaticoduodenectomy with cholecystectomy. Postoperative pathological examination confirmed primary moderately differentiated extrahepatic SCC of the bile duct.

**Conclusions:**

We report a rare case of primary pure SCC located in the pancreatic segment of the common bile duct, without adenocarcinoma components or metaplastic changes. This case underscores its rarity and diagnostic challenges, and highlights the critical role of histopathology and immunohistochemistry in confirming the diagnosis and excluding secondary tumors. Moreover, it broadens the clinicopathologic spectrum of extrahepatic bile duct malignancies and emphasizes that primary SCC should be considered in the differential diagnosis of distal biliary lesions mimicking pancreatic cancer.

## Introduction

Squamous cell carcinoma (SCC) of the biliary system is rare, accounting for <2% of biliary malignancies. Since its first report by Cabot in 1930, only a limited number of cases have been described. Cholangiocarcinoma (CCA) arises from the bile duct epithelium and may occur anywhere along the intrahepatic or extrahepatic bile ducts. Most bile duct malignancies are histologically adenocarcinomas, whereas SCC is exceedingly rare.

This report presents a rare case of SCC of the extrahepatic bile duct arising in the pancreatic segment that was clinically misdiagnosed as pancreatic cancer. Following surgery, the patient did not receive chemotherapy or radiotherapy. At 11 months of follow-up, no recurrence or metastasis was observed.

## Case presentation

A 70-year-old man with no history of malignancy presented with a one-month history of right upper quadrant abdominal pain and jaundice, reportedly worsening after meals. Laboratory tests showed a carbohydrate antigen 125 (CA125) level of 11.8 U/mL and a CA19–9 level of 223.50 U/mL. Total bilirubin was 235.63 μmol/L, with direct bilirubin of 134.67 μmol/L, and indirect bilirubin of 100.96 μmol/L.

**Figure 1:**
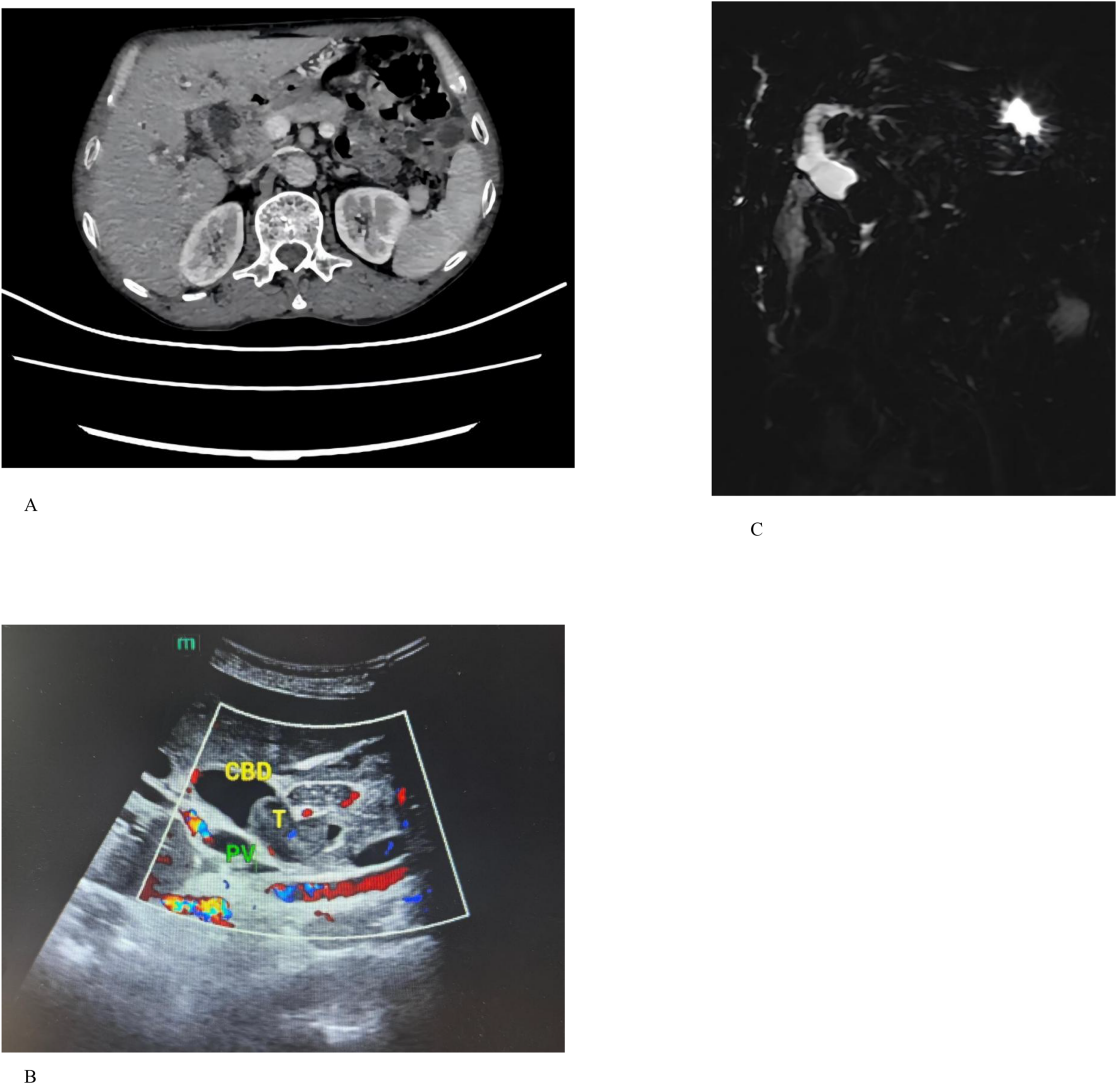
**(A)** CT examination reveals cholecystitis with dilation of the intrahepatic bile ducts, common hepatic duct, and proximal common bile duct; the middle and distal segments are poorly visualized. **(B)** Abdominal ultrasound demonstrates a space-occupying lesion in the common bile duct suggestive of pancreatic cancer, with associated intrahepatic and extrahepatic bile duct dilation. **(C)** MRCP demonstrates poor visualization of the gallbladder and cystic duct. Focal truncation of the pancreatic segment of the common bile duct (CBD) with an intraluminal filling defect is observed. The CBD, common hepatic duct, bilateral hepatic ducts, and intrahepatic bile ducts are dilated, without additional filling defects. The main pancreatic duct was not dilated.

Computed tomography (CT) revealed gallbladder stones, cholecystitis, and dilation of the intrahepatic bile ducts, common hepatic duct, and proximal common bile duct. The middle and distal segments were poorly visualized, and magnetic resonance cholangiopancreatography (MRCP) was recommended (**Figure 1A**). Abdominal ultrasound demonstrated a space-occupying lesion in the common bile duct, suggestive of pancreatic cancer with associated intrahepatic and extrahepatic ductal dilation (**Figure 1B**). Contrast-enhanced MRCP showed poor visualization of the gallbladder and cystic duct. The pancreatic segment of the common bile duct exhibited focal truncation with intraluminal filling defects. The common bile duct, common hepatic duct, bilateral hepatic ducts, and intrahepatic bile ducts were dilated, without intraluminal defects. The main pancreatic duct was not dilated (**Figure 1C**).

Due to obstructive jaundice, percutaneous transhepatic biliary drainage (PTBD) was performed on April 28, 2025. During the procedure, the intrahepatic ducts, their branches, the common hepatic duct, and the common bile duct were visualized, with distal common bile duct occlusion. Following drainage, the patient’s condition stabilized. On May 12, 2025, the patient underwent open radical pancreaticoduodenectomy with cholecystectomy. The resected specimen was submitted for postoperative gross pathological evaluation.

The greater curvature of the stomach measured 12.5 cm, the lesser curvature 5.5 cm, and the circumference 5.5 cm. The duodenum measured 26 cm in length with a diameter of 1.5–2 cm. A segment of pancreatic tissue measured 8.0 × 1.5 × 1.5 cm, extending along the greater curvature of the stomach and opposite the duodenal papilla into the adjacent small intestine. No mucosal abnormalities were observed. The common bile duct was opened from the proximal margin to the major duodenal papilla. It measured 3 cm in length with a circumference of 0.4–0.8 cm. A grayish-white, firm nodular lesion measuring 2.5 × 2.1 × 1 cm was identified within the duct. The cut surface was solid, grayish-white, and firm. The lesion was located 0.6 cm from the pancreatic resection margin and did not involve the ampulla. The gallbladder measured 11 × 3.5 × 1 cm, contained concentrated bile, and showed a flat mucosa with a wall thickness of 0.2 cm.

**Figure 2:**
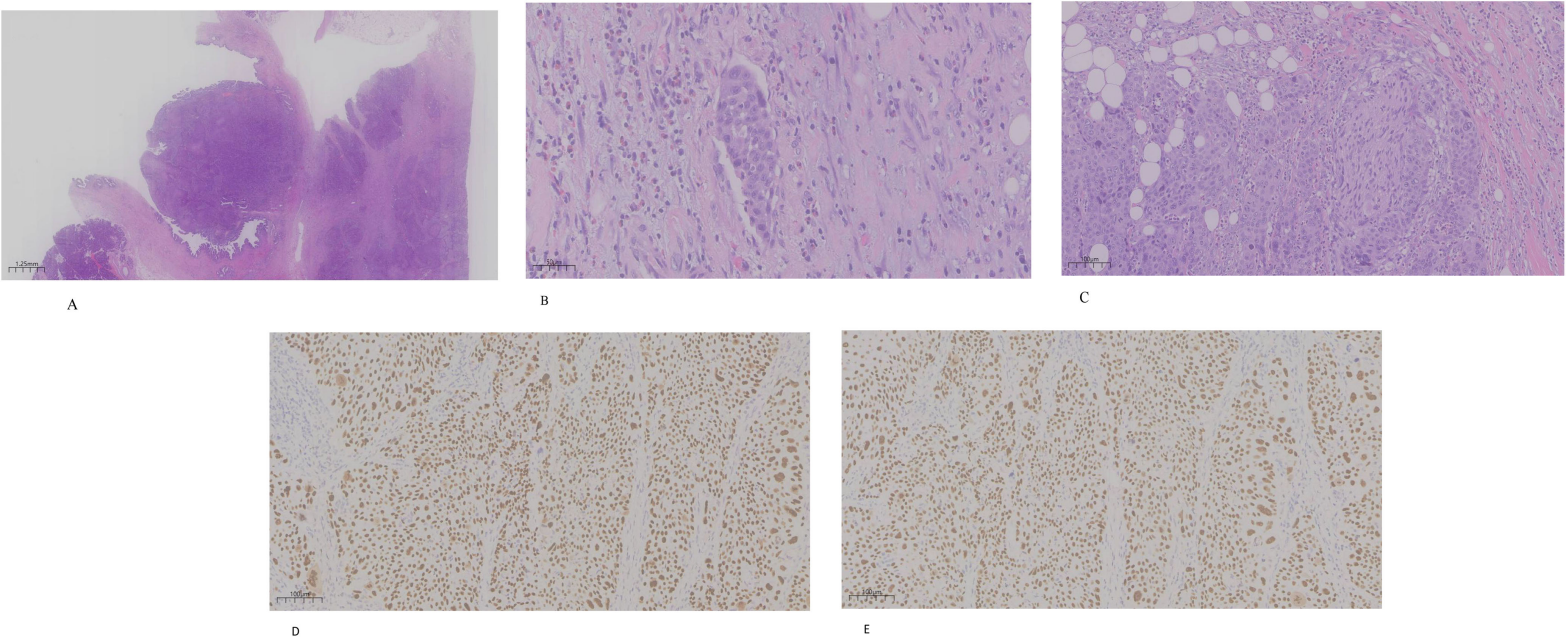
**(A)** Low-power view showing a tumor arising from the pancreatic segment of the CBD, protruding into the lumen and extending into the surrounding fibrous connective tissue (hematoxylin and eosin (H&E, ×0.63). **(B)** Medium-power view showing tumor cells within vascular lumina, consistent with lymphovascular invasion (H&E, ×100). **(C)** Medium-power view demonstrating perineural invasion by tumor cells (H&E, ×200). **(D)** Immunohistochemistry showing tumor cell positivity for p63 (EnVision, ×100). **(E)** Immunohistochemistry showing tumor cells positivity for p40 (EnVision, ×100).

Microscopically, the tumor arose from the pancreatic segment of the bile duct, protruded into the lumen, and infiltrated the periductal fibrous connective tissue (**Figure 2A**). Lymphovascular and perineural infiltration were present (**Figures 2B, C**). Immunohistochemistry (IHC) demonstrated positivity for pan-cytokeratin (CKpan), p63 (**Figure 2D**), and p40 (**Figure 2E**), and negativity for carcinoembryonic antigen (CEA), caudal-type homeobox 2 (CDX-2), cytokeratin 7 (CK7), CK19, and CK20. Complete embedding and sectioning of the tumor and bile duct revealed no adenocarcinoma component. Based on clinical history, examination findings, and auxiliary investigations, a pathological diagnosis of primary moderately differentiated SCC of the extrahepatic bile duct (pancreatic segment) was established. The gallbladder showed inflammatory changes without calculi.

## Discussion

Cholangiocarcinoma (CCA) is a relatively rare malignancy of the digestive tract that may arise anywhere along the biliary tree and is classified by primary site as intrahepatic, perihilar, or distal CCA (1). Adenocarcinoma is the predominant histological type, whereas SCC is extremely rare, accounting for <2% of biliary malignancies (2). The first case of bile duct SCC was reported by Cabot in 1930 (3), and only a limited number of cases have since been described. To our knowledge, this report represents a rare case and is the first reported instance of moderately differentiated SCC located in the pancreatic segment of the common bile duct, with diagnosis confirmed on the resected specimen.

Although the present case was diagnosed by postoperative surgical pathology, the optimal diagnostic approach for extrahepatic biliary strictures, particularly when rare tumors such as primary SCC are suspected, warrants discussion. Endoscopic ultrasound-guided tissue acquisition (EUS-TA) has emerged as a key modality for evaluating biliary and pancreatic masses. Notably, EUS-guided fine-needle biopsy (EUS-FNB) demonstrates superior diagnostic accuracy and sample adequacy compared with EUS-guided fine-needle aspiration (EUS-FNA), especially for solid pancreatic lesions (4). The ability of FNB to obtain core tissue specimens preserves architecture, which is essential for IHC characterization—a cornerstone in diagnosing primary biliary SCC, as in this case. The recent European Society of Gastrointestinal Endoscopy (ESGE) guideline recommends combining EUS-TA with ERCP-based tissue acquisition for patients with jaundice and distal extrahepatic biliary stricture without an identifiable pancreatic mass (5).

Furthermore, for indeterminate biliary strictures, cholangioscopy-guided biopsy is recommended in addition to standard ERCP-based techniques (5). Given that the tumor in this case was located in the pancreatic segment of the common bile duct and was predominantly intraductal, cholangioscopy could have enabled direct visualization and targeted biopsy, potentially allowing preoperative diagnosis. Although these advanced endoscopic techniques were not used, given the rarity of the condition and the eventual confirmation by surgical pathology, they may be valuable in similar future cases. We suggest that in patients with extrahepatic biliary strictures of unclear etiology, particularly when imaging reveals an intraductal lesion without a discrete mass, a stepwise approach incorporating EUS-FNB followed by cholangioscopy-guided biopsy (if inconclusive) should be considered to achieve a definitive preoperative diagnosis and guide management.

**Table 1** shows the reported literature on primary pure SCC of the bile duct. Reported cases showed a mean age of 54 years (range, 24–78 years) with a male predominance (approximately 64% of cases). Our case, involving an older male patient, is consistent with the findings. Extrahepatic CCA is rare, typically affecting older individuals (2), and is associated with a poor prognosis. Patients typically present with jaundice, abdominal pain, and generalized weakness, sometimes accompanied by weight loss. Clinical manifestations are similar to those of other bile duct malignancies, with jaundice and abdominal pain being the most frequent symptoms. Laboratory findings usually indicate elevated total and direct bilirubin, and cholestatic markers (6, 8). The present study suggests limited clinical utility of CA19–9 in choledochal SCC. Although CA19–9 is widely used in biliary tract malignancies, its levels fluctuated considerably in reported cases, with some patients showing marked elevations and others remaining within normal limits throughout the disease course (7–14). Imaging features of biliary SCC are non-specific and resemble those of adenocarcinoma, typically presenting as biliary dilation.

**Table 1:** Clinicopathological features of primary pure squamous cell carcinoma of the bile duct.

Case	Sex	Yeas	Location	Laboratory	Clinical symptoms	Tumor markers	Tumor size (cm)	Therapy	Follow-up (month)	Clinical outcomes
1 (6)	F	24	Common Bile Duct	total bilirubin of 8.1 mg/100ml, Alkaline phosphatase 161 IU/L	right upper quadrant and jaundice of two weeks	CEA9.1ng/ml	NM NR	Surgery +chemotherapy	8	Death
2 (7)	M	76	Common Bile Duct	Total bilirubin (312.1 µmol/, alkaline phosphatase 331 U/L	jaundice, dark urine, and clay-colored stools with epigastric pain.	CA19-9 728.5 U/mL	3	Surgery	6	Loss to follow-up
3 (8)	M	35	Common Bile Duct	total bilirubin 376 µmol/l,	epigastric pain, jaundice and general fatigue for one month.	CA19-9 and CEA normal limits	NR	Surgery +chemotherapy	1	Death
4 (9)	M	52	the hepatic caudate	total bilirubin level 7.70 μmol/L	a tumor of the hepatic caudate lobe that had been detected 14 days	AFP16.34 ng/mL, CA19-9 and CEA normal limits	4	Surgery +chemotherapy	15	Recurrence
5 (10)	F	77	junction of the cystic and common bile ducts	total bilirubin (2.8 mg/dL,alkaline phosphatase 773 U/L,	jaundice and general fatigue	CA199 and CEA normal limits	1.7	Surgery +chemotherapy	32	Death
6 (9)	M	78	Distal CBD	NR	jaundice	SCC 10.7 ng/mL , CEA, CA19–9 and DUPAN-2 were norma	3	Surgery +chemotherapy	10	no recurrence
7 (11)	M	67	Distal CBD	total bilirubin, 20.3mg/dL, alkaline phosphatase, 924U/L	yellow skin and dark urine	CA199125.1U/mL	1.7	Surgery	8	Death
8 (12)	F	32		total bili rubin 35.1 lmol/L , phosphatase level, 750 IU/L	developed abdominal discomfort 6 months,jaundice	CEA,CA199,AFPnormal	NR	Surgery +chemotherapy	6	Recurrence,liver metastasis
9 (13)	F	28	Common Bile Duct	NR	moderate right upper quadrant pain associated with jaundice	NR	NR	Surgery +radiotherapy	18	No recurrence or metastasis
10 (14)	M	64	Common Bile Duct	total bilirubin: 3.06 mg/dl	right upper-quadrant pain, jaundice, and weight loss.	CA1991300U/mL	13.5	Surgery +chemotherapy	2	Recurrence
Our case	M	70	Pancreatic segment bile duct	Total bilirubin: 235.63umol/L	right upper quadrant abdominal pain and jaundice	CA199223.50U/ml	2.5	Surgery	11	Recurrence

CBD, common bile duct; NR, not reported.

Histologically, normal biliary mucosa consists of a single layer of cuboidal epithelium without squamous cells (15). Therefore, adenocarcinoma is the predominant histological type of biliary malignancy (16), whereas SCC is exceedingly rare, and its pathogenesis remains unclear. One hypothesis proposes that chronic inflammation induces squamous metaplasia of the biliary epithelium, which subsequently progresses to carcinoma (17). SCC has also been reported in association with choledochal cysts (9, 18). Moreover, gallstones and ulcerative colitis have been linked to biliary malignancies (19). However, in the present case, no squamous metaplasia or choledochal cyst was identified histologically. The tumor may have originated from undifferentiated primitive pluripotent cells (20), although the exact pathogenesis remains unclear. Kohno et al. reported histological transition from adenocarcinoma to SCC and observed a higher growth rate of SCC, suggesting possible transformation from adenocarcinoma (3).

Because SCC of the bile duct lacks specific clinical features, imaging findings, and serum markers, diagnosis relies on histopathological examination, supported by IHC. In this case, the IHC profile showed CK5/6 and p40 positivity, with CK7 and CK20 negativity. Chest CT, esophagogastroduodenoscopy (EGD), and laryngoscopy excluded primary SCC of the lung, esophagus, and head and neck. Based on these findings and the absence of other primary sites, the tumor was considered a primary SCC of the common bile duct. A comprehensive sampling of the tumor and surrounding tissue is essential to identify any adenocarcinoma component. In this case, extensive examination revealed no such component, supporting the diagnosis of primary extrahepatic biliary SCC. Given the rarity of this entity, a detailed clinical history, including prior malignancies, is important; this patient had no history of cancer, further supporting a primary origin. For differential diagnosis, primary biliary SCC should be distinguished from hepatocellular carcinoma, intrahepatic CCA, and metastatic carcinoma. In particular, tumors arising in the pancreatic segment must be differentiated from pancreatic malignancies.

Molecular studies have reported alterations in *TP53*, *JAK2* amplification, and mutations in *FBXW7*, *CREBBP*, *CTCF*, *FAT1*, *MAGI2*, *MLL2*, and *NOTCH1* in biliary SCC (8, 21, 22). However, their associations with tumor invasion, prognosis, drug resistance, or potential targeted therapies remain to be clarified in larger studies.

Currently, surgical resection remains the primary treatment for CCA and is associated with improved survival. Adjuvant chemotherapy may further enhance outcomes. Commonly used chemotherapeutic regimens include GEMOX (gemcitabine plus oxaliplatin) or GP (gemcitabine plus cisplatin) (23). Other regimens, such as docetaxel combined with cisplatin and 5-fluorouracil (5-FU), or tegafur/gimeracil/oteracil (S-1) combined with cisplatin, may also be effective (24). Postoperative adjuvant chemotherapy with S-1 has been reported to prolong overall survival and reduce recurrence risk (24). Chemotherapy and radiotherapy have been applied as adjuvant treatment, for advanced metastatic disease, and for postoperative recurrence (24). Patients receiving adjuvant therapy appear to have better outcomes than those who do not. However, most reported cases lacked adjuvant therapy and long-term follow-up, limiting firm conclusions; multimodal treatment may offer improved survival. The role of radiotherapy remains unclear and has not been systematically evaluated in CCA. Targeted therapy and immunotherapy have shown efficacy in other malignancies, but their role in biliary SCC requires further investigation through clinical trials.

The prognosis of bile duct SCC may be worse than that of adenocarcinoma, with a reported mortality rate of up to 63.3% (8). This finding may reflect its rarity and the absence of standardized treatment strategies. In the present case, no adjuvant therapy was administered postoperatively, and the patient remained free of recurrence or metastasis at 11 months of follow-up. Due to limited case data, definitive conclusions regarding survival in extrahepatic CCA cannot be drawn. Reported survival durations range from 1 month (25) to 32 months (10).

## Conclusion

We report an extremely rare case of primary pure SCC located in the pancreatic segment of the common bile duct that was clinically misdiagnosed as pancreatic cancer. This case highlights its rarity and diagnostic challenges, as well as the critical role of histopathology and IHC in confirming the diagnosis and excluding secondary tumors. It also broadens the clinicopathologic spectrum of extrahepatic bile duct malignancies and underscores the need to consider primary SCC in the differential diagnosis of distal biliary lesions.

## Data Availability

The original contributions presented in the study are included in the article/supplementary material, further inquiries can be directed to the corresponding author/s.
